# Association of Clinical and Demographic Factors With the Severity of Palmoplantar Pustulosis

**DOI:** 10.1001/jamadermatol.2020.3275

**Published:** 2020-09-16

**Authors:** Natashia Benzian-Olsson, Nick Dand, Charlotte Chaloner, Zsuzsa Bata-Csorgo, Riccardo Borroni, A. David Burden, Hywel L. Cooper, Victoria Cornelius, Suzie Cro, Tejus Dasandi, Christopher E. M. Griffiths, Külli Kingo, Sulev Koks, Helen Lachmann, Helen McAteer, Freya Meynell, Ulrich Mrowietz, Richard Parslew, Prakash Patel, Andrew E. Pink, Nick J. Reynolds, Adrian Tanew, Kaspar Torz, Hannes Trattner, Shyamal Wahie, Richard B. Warren, Andrew Wright, Jonathan N. Barker, Alexander A. Navarini, Catherine H. Smith, Francesca Capon

**Affiliations:** 1 Department of Medical and Molecular Genetics, King's College London, London, United Kingdom; 2 Health Data Research UK, London, United Kingdom; 3 Department of Dermatology and Allergology, University of Szeged, Szeged, Hungary; 4 Humanitas Clinical and Research Center, IRCCS, Milan, Italy; 5 Department of Biomedical Sciences, Humanitas University, Milan, Italy; 6 Institute of Infection, Immunity and Inflammation, University of Glasgow, Glasgow, United Kingdom; 7 Portsmouth Dermatology Unit, Portsmouth Hospitals Trust, Portsmouth, United Kingdom; 8 Imperial Clinical Trials Unit, School of Public Health, Imperial College London, London, United Kingdom; 9 St John's Institute of Dermatology, King's College London, London, United Kingdom; 10 Dermatology Centre, National Institute for Health Research Manchester Biomedical Research Centre, University of Manchester, Manchester, United Kingdom; 11 Dermatology Clinic, Tartu University Hospital, Department of Dermatology, University of Tartu, Tartu, Estonia; 12 Centre for Molecular Medicine and Innovative Therapeutics, Murdoch and Perron Institute for Neurological and Translational Science, Murdoch University, Nedlands, Western Australia, Australia; 13 National Amyloidosis Centre, University College London, Royal Free Campus, London, United Kingdom; 14 The Psoriasis Association, Northampton, United Kingdom; 15 Psoriasis Center at the Department of Dermatology, University Medical Center, Schleswig-Holstein, Campus Kiel, Kiel, Germany; 16 Department of Dermatology, Royal Liverpool Hospitals, Liverpool, United Kingdom; 17 Translational and Clinical Research Institute, Newcastle University, Newcastle upon Tyne, United Kingdom; 18 Department of Dermatology and National Institute for Health Research Newcastle Biomedical Research Centre, Newcastle Hospitals NHS Foundation Trust, Newcastle upon Tyne, United Kingdom; 19 Department of Dermatology, Medical University of Vienna, Austria; 20 Department of Dermatology, University Hospital of North Durham, Durham; 21 The Dermatology Centre, Salford Royal NHS Foundation Trust, University of Manchester, Manchester Academic Health Science Centre, Manchester, United Kingdom; 22 Department of Dermatology, St Lukes Hospital, Bradford, United Kingdom; 23 Department of Dermatology & Allergy, University Hospital of Basel, Basel, Switzerland

## Abstract

**Importance:**

Although palmoplantar pustulosis (PPP) can significantly impact quality of life, the factors underlying disease severity have not been studied.

**Objective:**

To examine the factors associated with PPP severity.

**Design, Setting, and Participants:**

An observational, cross-sectional study of 2 cohorts was conducted. A UK data set including 203 patients was obtained through the Anakinra in Pustular Psoriasis, Response in a Controlled Trial (2016-2019) and its sister research study Pustular Psoriasis, Elucidating Underlying Mechanisms (2016-2020). A Northern European cohort including 193 patients was independently ascertained by the European Rare and Severe Psoriasis Expert Network (2014-2017). Patients had been recruited in secondary or tertiary dermatology referral centers. All patients were of European descent. The PPP diagnosis was established by dermatologists, based on clinical examination and/or published consensus criteria. The present study was conducted from October 1, 2014, to March 15, 2020.

**Main Outcomes and Measures:**

Demographic characteristics, comorbidities, smoking status, Palmoplantar Pustulosis Psoriasis Area Severity Index (PPPASI), measuring severity from 0 (no sign of disease) to 72 (very severe disease), or Physician Global Assessment (PGA), measuring severity as 0 (clear), 1 (almost clear), 2 (mild), 3 (moderate), and 4 (severe).

**Results:**

Among the 203 UK patients (43 men [21%], 160 women [79%]; median age at onset, 48 [interquartile range (IQR), 38-59] years), the PPPASI was inversely correlated with age of onset (*r* = −0.18, *P* = .01). Similarly, in the 159 Northern European patients who were eligible for inclusion in this analysis (25 men [16%], 134 women [84%]; median age at onset, 45 [IQR, 34-53.3] years), the median age at onset was lower in individuals with a moderate to severe PGA score (41 years [IQR, 30.5-52 years]) compared with those with a clear to mild PGA score (46.5 years [IQR, 35-55 years]) (*P* = .04). In the UK sample, the median PPPASI score was higher in women (9.6 [IQR, 3.0-16.2]) vs men (4.0 [IQR, 1.0-11.7]) (*P* = .01). Likewise, moderate to severe PPP was more prevalent among Northern European women (57 of 134 [43%]) compared with men (5 of 25 [20%]) (*P* = .03). In the UK cohort, the median PPPASI score was increased in current smokers (10.7 [IQR, 4.2-17.5]) compared with former smokers (7 [IQR, 2.0-14.4]) and nonsmokers (2.2 [IQR, 1-6]) (*P* = .003). Comparable differences were observed in the Northern European data set, as the prevalence of moderate to severe PPP was higher in former and current smokers (51 of 130 [39%]) compared with nonsmokers (6 of 24 [25%]) (*P* = .14).

**Conclusions and Relevance:**

The findings of this study suggest that PPP severity is associated with early-onset disease, female sex, and smoking status. Thus, smoking cessation intervention might be beneficial.

## Key Points

**Question:**

Are clinical and demographic factors associated with the severity of palmoplantar pustulosis?

**Findings:**

In a cross-sectional study of 203 patients in the UK, the Palmoplantar Pustulosis Psoriasis Area Severity Index score was significantly higher in women compared with men and in current smokers vs former and never smokers. Both of these findings were replicated in an independently ascertained, Northern European cohort including 159 patients.

**Meaning:**

The findings of this study suggest that smoking cessation interventions may be beneficial in patients with palmoplantar pustulosis and should be investigated in clinical studies.

## Teaser

This cross-sectional study examines factors that may contribute to the severity of symptoms in patients with palmoplantar pustulosis.

## Introduction

Palmoplantar pustulosis (PPP) is an uncommon pustular eruption affecting the palms and/or soles. It is observed in approximately 1:2000 individuals of European descent and 1:800 individuals of East Asian descent.^1^ The disease typically manifests in adulthood, with a median age of onset older than 45 years reported in most studies.^2^ Palmoplantar pustulosis shows a marked sex bias, with women accounting for 60% to 90% of affected individuals.^2,3^ The disease is also characterized by an association with cigarette smoking, with up to 90% of patients self-identifying as smokers at the time of diagnosis.^2,4,5,6^

The disease manifests with the eruption of sterile, neutrophil-filled pustules on the palms and soles. The lesions, which can occur on a background of normal or inflamed skin, are persistent (>3 months), painful, and disabling, and can be accompanied by fissures, pruritus, and a burning sensation.^7^ Comorbidities are also common, as affected individuals are at increased risk of psoriasis vulgaris, psoriatic arthritis, and autoimmune thyroid disease.^8^

While PPP can profoundly impact quality of life, the factors underlying variable disease severity have not been investigated. The rarity of the condition has hindered the ascertainment and characterization of adequately powered data sets. In this context, the objective of our study was 2-fold: to evaluate the features of PPP in 2 independent patient cohorts and to examine whether PPP severity is influenced by sex and smoking status—the 2 most well-established risk factors for the disease.^1^ Given that symptoms typically manifest in adulthood, we also sought to examine whether the presentation of PPP is more severe in early-onset cases.

## Methods

### Patients

This study was carried out in accordance with the principles of the Declaration of Helsinki^9^ and with the approval of the participating institutions’ ethics committees. The present study was approved by London Bridge Research ethics committee (London, UK) and Kantonale Ethikkommission (Zurich, Switzerland). All patients granted their informed consent in writing; participants did not receive financial compensation. This study followed the Strengthening the Reporting of Observational Studies in Epidemiology (STROBE) reporting guideline for cross-sectional studies. The present study was conducted from October 1, 2014, to March 15, 2020.

The UK resource included 203 unrelated and prospectively ascertained patients. Forty-two patients were recruited between 2016 and 2019 through Anakinra in Pustular Psoriasis, Response in a Controlled Trial (APRICOT).^10^ The remaining 161 patients were enrolled between 2016 and 2020 through the sister research study Pustular Psoriasis, Elucidating Underlying Mechanisms (PLUM).^11^ A total of 23 dermatology centers located across the UK were involved in the recruitment.

The Northern Europe resource included 193 unrelated patients. Affected individuals were mostly enrolled between 2014 and 2017 through 3 centers affiliated with the European Rare and Severe Psoriasis Expert Network (ERASPEN). These centers were in the dermatology departments of the Medical University of Vienna, Austria (n = 100), Tartu University, Estonia (n = 57), and University Medical Centre Schleswig-Holstein, Campus Kiel, Germany (n = 31). The remaining 5 patients were recruited outside the main reference centers by clinicians who provided individual cases to the ERASPEN Consortium.

Pustular psoriasis was always diagnosed by a dermatologist, based on clinical examination and/or the ERASPEN consensus criteria.^7^ The observation of sterile, macroscopically visible pustules on palms or soles was the main inclusion criterion. Conversely, the presence of pustules restricted to the edges of psoriatic plaques represented an exclusion criterion. Individuals with concomitant generalized pustular psoriasis or concomitant acrodermatitis continua of Hallopeau were also excluded from the study, given that lesions affecting nails or nonacral skin are deemed incompatible with a diagnosis of PPP.^12^

Clinical information and key demographics were collated using a standardized case report form, shared by all centers. In the UK cohort, disease severity was measured using the Palmoplantar Pustulosis Area Severity Index (PPPASI)^13^ and the Dermatology Life Quality Index (DLQI) (eFigure 1 in the Supplement). The PPPASI measures severity of the disease with scores from 0 (no sign of disease) to 72 (very severe disease). The DLQI measures quality of life with scores ranging from 0 to 30, with higher scores indicating greater impairment. In the Northern European cohort, patients were assessed with the Physician Global Assessment (PGA), which has been shown to correlate with the PPPASI.^14^ The individuals who recorded clinical data and measured disease severity (reporting dermatologists or trained research nurses) were blinded to the study objectives.

### Statistical Analysis

Given that different scoring systems were used in the UK and Northern European cohorts, the 2 data sets were analyzed separately. The quantitative PPPASI and DLQI measures obtained in the UK cohort were analyzed using the Mann-Whitney test for binary variables, such as sex, or the Kruskal-Wallis test for categorical variables, such as smoking status. The correlation between PPPASI or DLQI findings and age at onset was assessed using the Spearman rank correlation coefficient. To account for the confounding effects of therapeutic intervention, statistical significance was confirmed by regression analysis. The PPPASI and DLQI values were normalized (square root transformation) and analyzed vs sex, age of onset, or smoking status, using treatment as a covariate.

To maximize statistical power, the categorical PGA scores recorded in the Northern European data set were dichotomized into clear to mild (including PGA-0 [clear], PGA-1 [almost clear], and PGA-2 [mild]) and moderate to severe (including PGA-3 [moderate] and PGA-4 [severe]). The 2 groups were then compared using the Fisher exact test. Given that the purpose of the PGA analysis was to replicate results showing statistical significance in the UK cohort, *P* values were computed based on a 1-tailed distribution. As the number of individuals receiving systemic treatment was relatively small (n = 25 from the Medical University of Vienna and 9 from Tartu University), the confounding effect of therapeutic intervention was addressed by excluding these cases from downstream analyses.

Individuals for whom information on smoking status or age at onset was missing (Table) were excluded from the relevant analyses. All statistical tests were implemented in R, version 3.6.1 (R Project for Statistical Computing). *P* values <.05 were considered statistically significant.

**Table.** Features of Study Cohorts

Cohort	United Kingdom	Northern Europe
Demographic characteristics^a^		
Women, No. (%)	160/203 (79)	161/193 (83)
Men, No. (%)	43/203 (21)	32/193 (17)
Age at onset, median (IQR), y^b^	48 (38-59)	45 (33-54)
Family history of psoriasis vulgaris, No. (%)	65/203 (32)	33/193 (17)
Family history of pustular psoriasis, No. (%)	9/203 (4)^c^	10/93 (11)^d^
Smoking status, No. (%)		
Current	90/203 (44)	124/193 (64)
Former	88/203 (43)	36/193 (19)
Never	23/203 (11)	28/193 (15)
Unknown	2/203 (1)	5/193 (3)
Clinical presentation		
Disease duration, median (IQR), y^b^	6 (2-14)	16 (10-20)^d^
Nail involvement, No. (%)	65/203 (32)	64/193 (33)
Concurrent psoriasis vulgaris, No. (%)	66/203 (33)	22/193 (11)
Concurrent psoriatic arthritis, No. (%)	20/203 (10)	17/193 (9)
Severity		
PPPASI score, median (IQR)^e^	8.2 (2.2-15.6)	NA
DLQI score, median (IQR)^f^^,^^g^	10 (3.3-16)	NA
On systemic treatment, No. (%)^h^	78/203 (38)	34/193 (18)
PGA score, No. (%)^i^	NA	
Clear/mild (0-2)		120/193 (62)
Moderate/severe (3-4)		73/193 (38)
Comorbid disease, No. (%)		
Asthma	25/203 (12)	5/93 (5)^d^
Depression	31/203 (15)	28/193 (15)
Diabetes	26/203 (13)	29/193 (15)
Hypertension	41/203 (20)	58/193 (30)
Autoimmune thyroid disease	14/203 (7)	25/193 (13)
Obesity^j^	60/150 (40)^k^	51/193 (26)

Abbreviations: DLQI, Dermatology Life Quality Index; IQR, interquartile range; NA, not applicable; PGA, Physician Global Assessment; PPPASI, Palmoplantar Pustular Psoriasis Area Severity Index.

^a^ All study participants were of European descent.

^b^ Data not available for 11 UK cases and 1 Northern European case.

^c^ One patient had a family history of both psoriasis vulgaris and pustular psoriasis.

^d^ Information not available for the 100 patients recruited in Vienna, Austria.

^e^ PPPASI measures severity with scores from 0 (no sign of disease) to 72 (very severe disease).

^f^ Data not available for 8 UK cases.

^g^ DLQI measures quality of life with scores from 0 to 30; higher scores indicate greater impairment.

^h^ On systemic treatment at the time of recruitment or the preceding 4 weeks.

^i^ PGA measures severity as 0 (clear), 1 (almost clear), 2 (mild), 3 (moderate), and 4 (severe).

^j^ Body mass index greater than 30 (calculated as weight in kilograms divided by height in meters squared).

^k^ Data not available for 53 UK cases.

## Results

### Patient Cohorts

The features of the UK and Northern European cohorts are summarized in the Table. Among the 203 UK patients (43 men [21%]; 160 women [79%]; median age at onset, 48 [interquartile range (IQR), 38-59] years). The Northern European cohort comprised 193 patients (32 men [17%]; 161 women [83%]; median age at onset, 45 [IQR, 33-54] years). Of these, 159 patients were available for analysis (25 men [16%]; 134 women [84%]; median age at onset, 45 [IQR, 34-53.3] years). All patients were of European descent. The percentage of women (>75%), median age of onset (≥45 years), and prevalence of current and former smokers (>80%) were comparable in the 2 data sets. Prominent nail involvement was observed in both study populations, with more than 30% of patients presenting with at least 1 of the following: pustules involving the nail apparatus, subungual hyperkeratosis, permanent nail loss, and nonpustular nail dystrophy.

Concurrent psoriasis vulgaris was observed in substantial numbers of study participants (66/203 [33%] UK patients and 22/193 [11%] Northern European cases), while psoriatic arthritis was reported in fewer patients from both cohorts (20/203 [10%] UK cases and 17/193 [9%] Northern European individuals).

Autoimmune thyroid disease was reported in several affected individuals from both data sets (14 of 203 [7%] of UK cases and 25 of 193 [13%] of Northern European patients). The prevalence of obesity (60 of 150 [40%] in the UK data set and 51 of 193 [26%] in the Northern European sample) was comparable to that observed among the overall population of British (36%),^15^ German (25%),^16^ and Estonian (21%)^16^ adults. In keeping with this observation, no correlation between the body mass index of patients with PPP and their PPPASI was noted (Spearman r = 0.03, *P* = .61).

While different scoring systems were used in the 2 cohorts, both included a substantial proportion of individuals with severe PPP, reflecting ascertainment in hospital settings. Specifically, 84 of 203 UK patients (41%) had a PPPASI score greater than 10 and 73 of 193 of their Northern European counterparts (38%) had a PGA score greater than or equal to 3 (Table).

### Disease Severity

**Figure 1.**
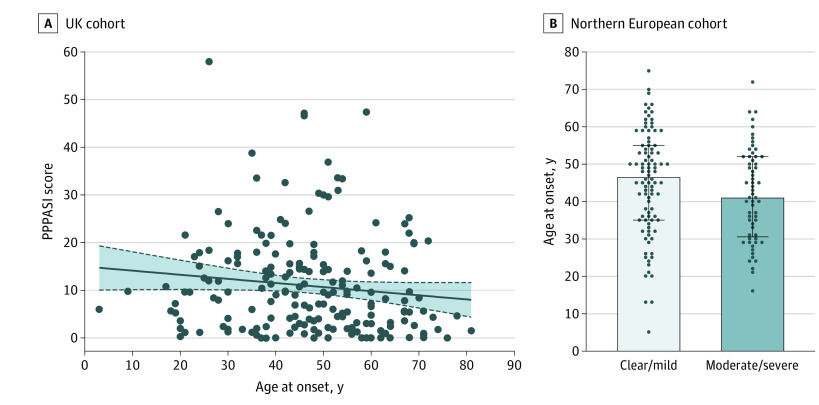
Association Between Disease Severity and Age of Onset A, In the UK cohort, the Palmoplantar Pustulosis Psoriasis Area Severity Index (PPPASI) score was inversely correlated with age of onset (*r* = −0.18, *P* = .01). Regression lines are plotted with their 95% CIs (gray areas). B, In the Northern European sample, age of onset was significantly lower among patients with moderate-to-severe disease. Data are presented as median (interquartile range). *P* < .05 per Mann-Whitney test. PPPASI measures severity with scores from 0 (no sign of disease) to 72 (very severe disease).

Among UK patients, age at onset was inversely correlated with the PPPASI score (*r* = −0.18, *P* = .01) (Figure 1A), although not with DLQI score (*r* = −0.08, *P* = .21). The association with the PPPASI score remained significant when the confounding effect of systemic treatment was taken into account by linear regression (*P* = .04) (eTable in the Supplement). In keeping with these findings, the analysis of the Northern European cohort revealed that the median age at onset was lower in patients with moderate to severe PGA (41 [IQR, 30.5-52] years) compared with those with clear to mild PGA (46.5 [IQR, 35.0-55.0] years) (*P* = .04) (Figure 1B). Thus, severe PPP appeared to be associated with early disease onset.

**Figure 2.**
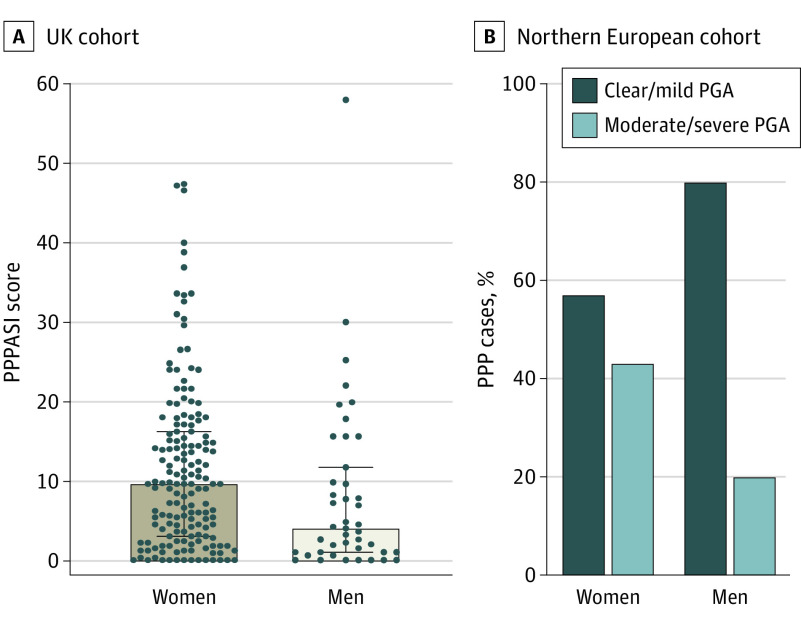
Disease Severity Scores in Women and Men A, In the UK cohort, Palmoplantar Pustulosis Psoriasis Area Severity Index (PPPASI) scores were significantly higher in women than men. Data are presented as median (interquartile range). *P* < .01 per Mann-Whitney test. B, In the Northern European sample, the proportion of individuals with moderate to severe disease was significantly elevated in women compared with men. *P* < .05 per Fisher exact test. Physician Global Assessment (PGA) measures severity as 0 (clear), 1 (almost clear), 2 (mild), 3 (moderate), and 4 (severe). PPPASI measures severity with scores from 0 (no sign of disease) to 72 (very severe disease). PPP indicates palmoplantar pustulosis.

In the UK sample, the median PPPASI score was higher in women (9.6 [IQR, 3.0-16.2]) compared with men (4.0 [IQR, 1.0-11.7]) (*P* = .01) (Figure 2A). The same applied to the median DLQI (women: 10.5 [IQR, 4.3-17] vs men: 4 [IQR, 1-9]; *P* = 8.2 × 10^−5^) (eFigure 2 in the Supplement). Both associations were confirmed when systemic treatment was included in a linear regression model (*P* = .03 for PPPASI, *P* < .001 for DLQI) (eTable in the Supplement). In agreement with these observations, analysis of the Northern European data set revealed that moderate to severe PPP was more prevalent among women (57 of 134 [43%]) compared with men (5 of 25 [20%]) (*P* = .03) (Figure 2B). Thus, PPP severity appeared to be associated with the sex of the patients in both the UK and Northern European study populations.

**Figure 3.**
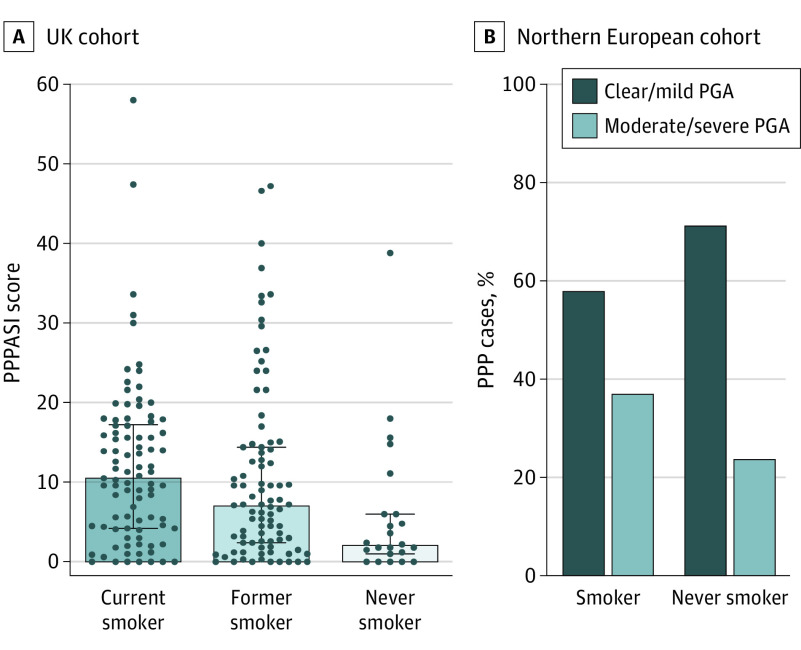
Disease Severity Scores in Current, Former, and Never Smokers A, In the UK cohort, Palmoplantar Pustulosis Psoriasis Area Severity Index (PPPASI) scores are highest in current smokers, intermediate in former smokers and lowest in never smokers. Data are presented as median (interquartile range). *P* < .01 per Kruskal-Wallis test. B, In the Northern European sample, the proportion of individuals with moderate to severe disease was elevated in current and former smokers compared with never smokers. Physician Global Assessment (PGA) measures severity as 0 (clear), 1 (almost clear), 2 (mild), 3 (moderate), and 4 (severe). PPPASI measures severity with scores from 0 (no sign of disease) to 72 (very severe disease). PPP indicates palmoplantar pustulosis.

Among the UK patients, the median PPPASI score was highest in current smokers (10.7 [IQR, 4.2-17.5]), intermediate in former smokers (7 [IQR, 2.0-14.4]), and lowest among nonsmokers (2.2 [IQR, 1-6]) (*P* = .003) (Figure 3A). Comparable findings were obtained when the median DLQI scores were analyzed (current smokers: 10 [IQR, 4.8-16.3] vs former smokers: 9 [IQR, 3-17] vs nonsmokers: 5 [IQR, 1-10.8]) (*P* = .04) (eFigure 3 in the Supplement). Both associations could be replicated when the effects of systemic treatment were incorporated into a linear model (*P* = .005 for PPPASI, *P* = .04 for DLQI) (eTable in the Supplement). The percentage of current smokers (men: 18/43 [42%] and women: 72/160 [45%]) and former smokers (men: 19/43 [44%] and women: 69/160 [43%]) was comparable in the 2 sexes. Moreover, multivariable regression modeling found no evidence that the effect of smoking differed by sex (data available from the authors).

While the analysis of the smaller Northern European data set did not yield statistically significant results, we observed a similar trend toward increased disease severity in smokers. Moderate to severe PPP was more frequent among current and former smokers (51 of 130 [39%]) than nonsmokers (6 of 24 [25%]) (*P* = .14) (Figure 3B).

## Discussion

To our knowledge, this is the first systematic study of the factors associated with PPP severity. This study builds on previous work from our network, which enabled the definition of consensus diagnostic criteria for PPP^7^ and suggested that the disease is genetically different from other forms of pustular psoriasis.^2^

Our investigation noted key epidemiologic features of PPP, such as the late age of onset and sex bias (male-female ratios were >1:3.5 in both cohorts). Psoriasis vulgaris concurrence, which is frequently reported in PPP, was also observed in the 2 data sets. While the prevalence of psoriasis vulgaris in the 2 cohorts was consistent with published estimates (14%-61%),^17^ the number of individuals with both PPP and psoriasis vulgaris was too small for subgroup analyses, and the use of different scoring systems prevented us from merging the UK and Northern European data sets. Conversely, the study of the entire resource highlighted aspects of PPP that, to our knowledge, had not been systematically investigated before.

We observed nail involvement in approximately one-third of affected individuals. Subungual pustulation was reported in a similar fraction of cases in a small UK study,^18^ suggesting that nail abnormalities are a consistent feature of PPP.

We also report substantial comorbidity with psoriatic arthritis, which was present in both cohorts at a frequency greater than 9%. This percentage exceeds the prevalence of the disease in the general population (0.1%-0.3%).^19^

Obesity was relatively uncommon, affecting only one-third of all study participants. This level contrasts with findings obtained in psoriasis vulgaris studies, where the association with obesity is well established^20^ and up to 42% of individuals with severe disease have a body mass index greater than 30.^21^

Overall, these findings suggest that PPP is part of the psoriasis spectrum because the substantial comorbidity with psoriasis vulgaris and psoriatic arthritis points to shared pathogenic pathways. At the same time, the distinctive demographics of PPP suggest the involvement of risk factors that are specific to this disease. For example, the female bias that characterizes PPP is not observed in palmoplantar psoriasis.^22^ Likewise, psoriasis vulgaris affects both sexes equally^23^ and occurred with comparable frequency in the male (15/28 [35%]) and female (51/160 [32%]) patients examined in this study (*P* = .72).

Our analysis of PPPASI and PGA scores suggests that PPP severity is higher in women vs men. Further experimental studies will be required to dissect the causes of this phenomenon. These sources may involve genetic modifiers or hormonal imbalances that could be targeted for disease treatment.

Our study also noted an association between cigarette smoking and disease severity that was statistically significant in the UK cohort (*P* < .01), where PPPASI values were highest in smokers, intermediate in former smokers, and lowest in nonsmokers. This observation suggests a clinically relevant dosage effect that may be validated and refined by analyzing pack-year data in further patient resources.

Smoking cessation is sometimes applied to the management of PPP and was found to be beneficial in a pilot study.^24,25^ In this context, our findings suggest that smoking cessation should be systematically investigated in adequately powered trials.

### Limitations

This study has limitations. The setting was exclusively based in secondary and tertiary referral centers, where the proportion of patients with severe PPP and the burden of comorbid disease may be higher than in other settings. Thus, the potential for ascertainment bias limits the generalizability of our findings.

Different measures of disease severity were used in the UK (DLQI and PPPASI) and Northern European cohorts (PGA). Although these scales are widely used in clinical practice, our results suggest that the categorical nature of the PGA tool affected the statistical power of the Northern European cohort and limited our ability to apply correlation-based methods. Thus, quantitative measurements, such as the PPPASI, or even more-sensitive methods, such as machine-learning–based pustule counts, should be considered the standard for studies of PPP severity.

## Conclusions

The findings from this cross-sectional study note the benefits of multicenter collaboration and standardized data collection in the analysis of rare skin diseases. The study also suggests that PPP symptoms are particularly severe in patients with early-onset disease, women, and current smokers. The association between the severity of the disease and smoking will need to be replicated in further data sets; however, the increased severity suggests that smoking cessation interventions may benefit the treatment of PPP.

## Notes

Supplement. **eFigure 1.** Correlation Between PPPASI and DLQI **eFigure 2.** DLQI Scores in Females and Males **eFigure 3.** DLQI Scores in Current, Former and Never Smokers **eTable.** Summary of Regression Analysis Results
