# Supplementary material for: Association of Clinical and Demographic Factors With the Severity of Palmoplantar Pustulosis
Source: JAMA Dermatol. 2020 Sep 16;156(11):1–8. doi: 10.1001/jamadermatol.2020.3275 (PMC7495329; doi:10.1001/jamadermatol.2020.3275)
Supplement: Supplement. — eFigure 1. Correlation Between PPPASI and DLQI eFigure 2. DLQI Scores in Females and Males eFigure 3. DLQI Scores in Current, Former and Never Smokers eTable. Summary of Regression Analysis Results [file jamadermatol-e203275-s001.pdf]

Supplementary Online Content

Benzian-Olsson N, Dand N, Chaloner C, et al; the ERASPEN Consortium and the APRICOT and PLUM Study Team. Association of clinical and demographic factors with the severity of palmoplantar pustulosis. *JAMA Dermatol*. Published online September 16, 2020. doi:10.1001/jamadermatol.2020.3275

eFigure 1. Correlation Between PPPASI and DLQI

eFigure 2. DLQI Scores in Females and Males

eFigure 3. DLQI Scores in Current, Former and Never Smokers

eTable. Summary of Regression Analysis Results

This supplementary material has been provided by the authors to give readers additional information about their work.

© 2020 Benzian-Olsson N et al. *JAMA Dermatology*.

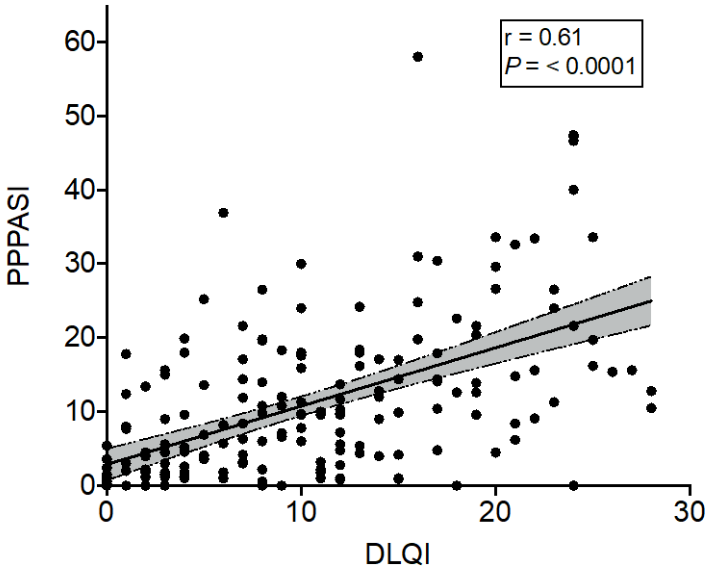

eFigure 1. Correlation between PPPASI and DLQI. Each point represents a patient. Regression lines are plotted with their 95% confidence intervals (grey areas).

© 2020 Benzian-Olsson N et al. *JAMA Dermatology*.

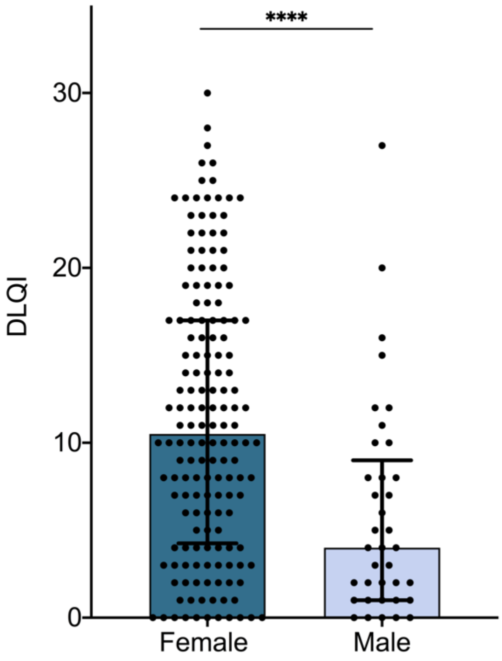

eFigure 2. DLQI scores in females and males. In the UK cohort, PPPPASI scores are significantly higher in females than males. Data are presented as median +/- IQR. ****P<0.0001 (Mann-Whitney test).

© 2020 Benzian-Olsson N et al. *JAMA Dermatology*.

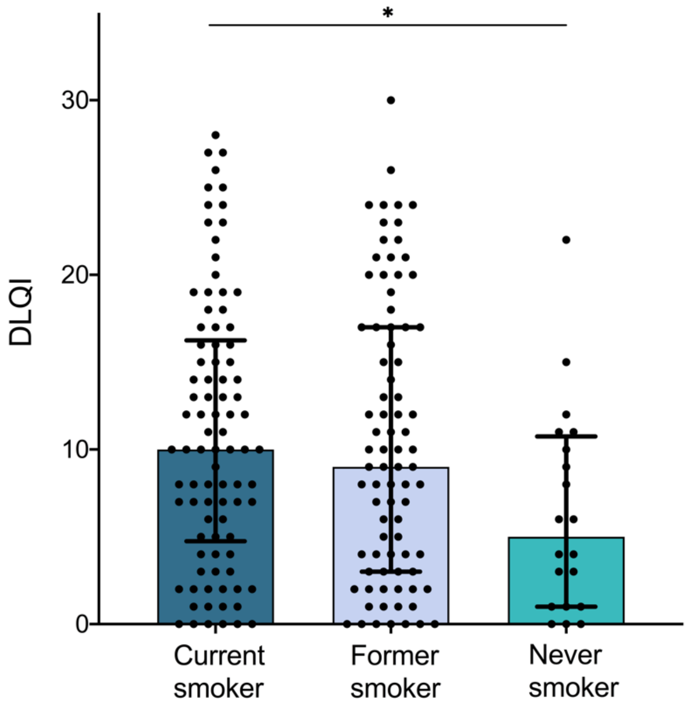

eFigure 3. DLQI scores in current, former and never smokers. In the UK cohort, DLQI scores are highest in current smokers, intermediate in former smokers and lowest in never smokers. Data are presented as median +/- IQR. **P*<0.05 (Kruskal-Wallis test).

© 2020 Benzian-Olsson N et al. *JAMA Dermatology*.

eTable: Summary of effect sizes observed by regression analysis

		95% CI	
Analysis	*Beta (reference category)*[a]	*Lower bound*	*Upper bound*
Age of onset vs PPPASI	-0.016	-0.03	-0.0008
Sex vs PPPASI	0.60 (male)	0.05	1.14
Smoking status vs PPASI	1.21 (never smoker)[b]<br>1.05 (never smoker)[c]	0.48<br>0.31	1.95<br>1.79
Sex vs DLQI	1 (male)	0.5	1.5
Smoking status vs DLQI	0.84 (never smoker)[b]<br>0.79 (never smoker)[c]	0.16<br>0.1	1.53<br>1.48

Systemic treatment was included as a co-variate in all analyses. [a]For categorical variables; [b]Smokers vs never smokers; [c]Former vs never smokers. CI: confidence intervals.

© 2020 Benzian-Olsson N et al. *JAMA Dermatology*.
